# Targeting long non-coding RNA DANCR inhibits triple negative breast cancer progression

**DOI:** 10.1242/bio.023135

**Published:** 2017-07-31

**Authors:** Sha Sha, Dongya Yuan, Yuejun Liu, Baosan Han, Nanbert Zhong

**Affiliations:** 1 Department of Medical Genetics, Peking University Health Science Center, Beijing, 100038 China; 2 Department of Medical Science, Xizang Minzu University, Xianyang, Shaanxi Province, 712082 China; 3 Department of Immunology and Microbiology, Xizang Minzu University, Xianyang, Shaanxi Province, 712082 China; 4 Department of General Surgery, Haifushan Hospital, Weifang, Shandong, 262605 China; 5 Department of General Surgery, Shanghai Jiaotong University Xinhua Hospital, Shanghai, 200240 China

**Keywords:** Triple negative breast cancers (TNBC), Long non-coding RNA, DANCR, Cancer stem cell

## Abstract

Triple negative breast cancer (TNBC) is non-responsive to conventional anti-hormonal and Her2-targeted therapies, making it necessary to identify new molecular targets for therapy. Long non-coding RNA anti-differentiation ncRNA (lncRNA DANCR) was identified participating in carcinogenesis of hepatocellular carcinoma, but its expression and potential role in TNBC progression is still unclear. In the present study, our results showed that DANCR expression was increased in TNBC tissues compared with the adjacent normal tissues using quantitative real-time PCR (qRT-PCR) in 63 TNBC specimens. Patients with higher DANCR expression correlated with worse TNM stages as well as a shorter overall survival (OS) using Kaplan–Meier analysis. When the endogenous DANCR was knocked-down via specific siRNA, cell proliferation and invasion were decreased obviously in the MDA-MB-231 cells. *In vivo* xenograft experiments showed that knockdown of the DANCR in MDA-MB-231 cells reduced the tumor growth significantly. Furthermore, a compendium of TNBC cancer stem cell markers such as CD44, ABCG2 transporter and aldehyde dehydrogenase (ALDH1) were greatly downregulated in the MDA-MB-231 cells with DANCR knockdown. Molecular mechanistic studies revealed that knockdown of DANCR was associated with increased binding of EZH2 on the promoters of CD44 and ABCG2, and concomitant reduction of expression of these genes suggested that they may be DANCR targets in TNBC. Thus, our study demonstrated that targeting DANCR expression might be a viable therapeutic approach to treat triple negative breast cancer.

## Abstract (precis)

**Summary:** Targeting LncRNA DANCR obstructs triple negative breast cancer progression by down-regulating cancer stem cell marker CD44 and ABCG2.

## INTRODUCTION

Developments in clinical treatment strategy, including the foundation of endocrine therapy and human epidermal growth factor receptor 2 (Her2)-targeted therapy, have improved the survival levels of breast cancer patients. However, triple negative-breast cancer (TNBC), which is characterized by the lack of an estrogen receptor (ER), progesterone receptor (PR), and Her2 overexpression, could not benefit from both endocrine therapy and Her2-targeted therapy (Agrawal and Mayer, 2014). TNBC high rates of recurrence and metastasis have been associated, in part, with a subpopulation of breast cancer stem-like cells (CSCs) that are resistant to conventional therapies, therefore one possible approach to achieve this therapeutic goal is to target the cancer stem cell self-renewal (Fang et al., 2016). CSCs are defined as a population of tumor-initiating or propagating cells possessing the ability to self-renew and differentiate (Kai et al., 2010), a compendium of markers such as CD44 high/CD24 low, and increased expression of the ABCG2 transporter and increased aldehyde dehydrogenase (ALDH1), have been associated with these cells (Jin et al., 2016).

Long non-coding RNAs (lncRNAs) are now recognized as a major component of the human transcriptome, but the vast majority of these molecules remain to be functionally annotated (Mattick and Rinn, 2015). Recently, many studies have shown that lncRNAs were frequently dysregulated in various cancers and have multiple functions in a wide range of biological processes, such as cell proliferation, cell apoptosis, cell cycle arrest and cell migration and invasion (Gutschner and Diederichs, 2012; Shen et al., 2015; Lv et al., 2016). These studies offer helpful information for understanding the initiation and development mechanisms of TNBC comprehensively, and suggest potential biomarkers for diagnosis or therapy targets for clinical treatment.

In recent years, plenty of reports have demonstrated that lncRNAs function as crucial regulators in TNBC development and progression. For instance, Pickard et al. showed that lncRNA GAS5 promotes apoptosis, and its expression is downregulated in breast cancer (Pickard and Williams, 2016). Zhang et al. identified blocking lncRNA LINP1 increases the sensitivity of the tumor-cell response to radiotherapy in breast cancer (Zhang et al., 2016). Jadaliha et al. demonstrated that MALAT1 facilitates cell proliferation, tumor progression and metastasis of TNBC cells despite having a comparatively lower expression level than ER or HER2-positive breast cancer cells (Jadaliha et al., 2016). Though the overall pathophysiological function of lncRNAs in TNBC remains to be unknown by now, previous studies strongly suggested that lncRNAs could be potential therapeutic targets in TNBC.

Given the importance of lncRNAs in TNBC, in the present study, we investigated the expression level of lncRNA differentiation antagonizing non-protein coding RNA (DANCR) (Kretz et al., 2012) in TNBC tissues and adjacent non-tumor tissues, as well as the association of lncRNA DANCR with clinicopathological characteristics and outcome of the TNBC patients. Moreover, we determined whether DANCR regulated cell proliferation, migration and invasion of TNBC. Furthermore, we identified the potential targets for CSC self-renewal and predicted their possible biological functions. Our results provide evidence for using the DANCR siRNA as a potential effective agent that targets CSCs in TNBC treatment.

## RESULTS

### DANCR knockdown decreased proliferation and invasion of TNBC cells

**Fig. 1.**
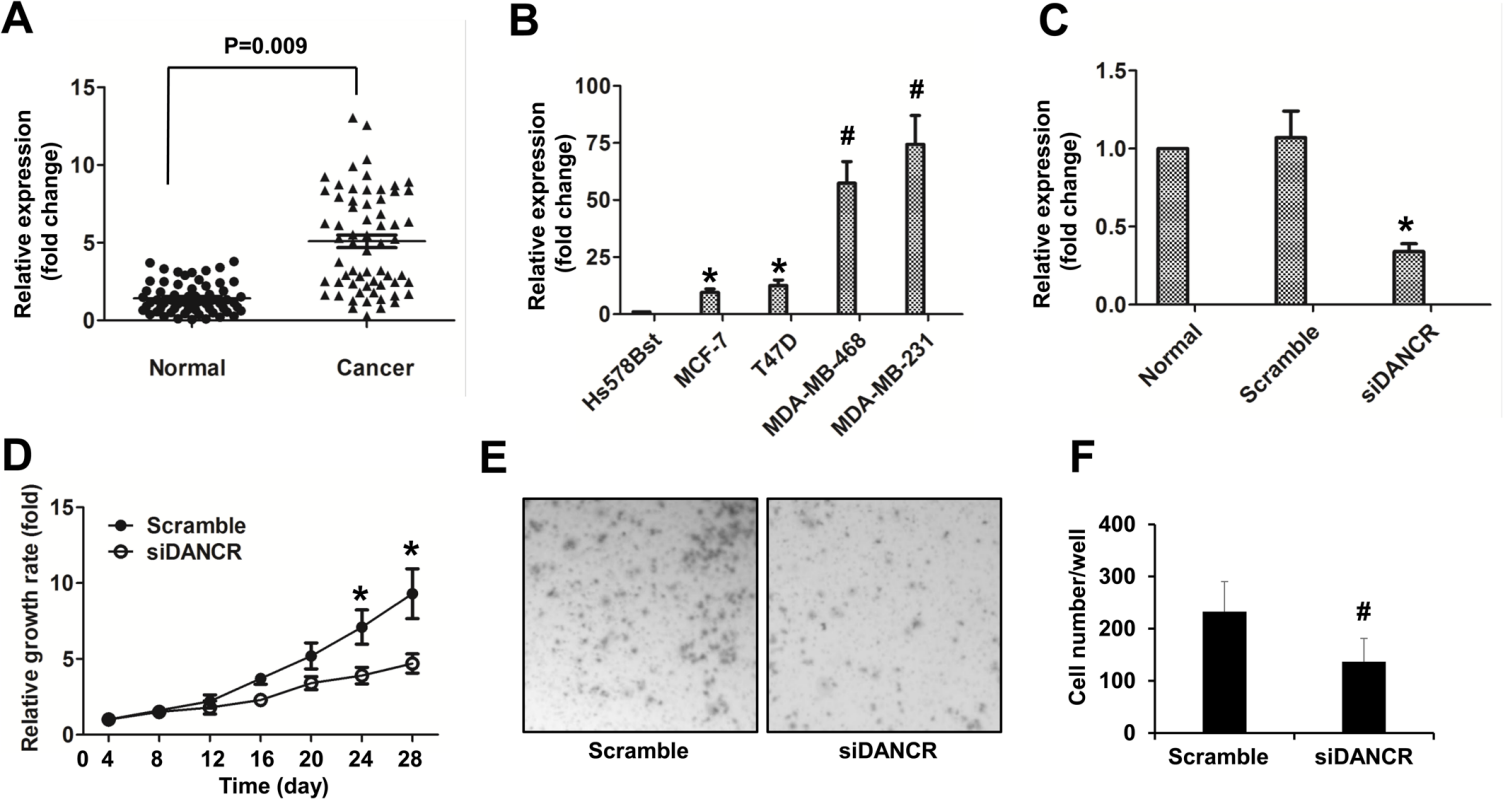
**DANCR knockdown decreased proliferation and invasion of TNBC cells.** (A) LncRNA DANCR expression levels assessed by qRT-PCR in TNBC tissue and adjacent non-tumor tissues. LncRNA DANCR expression levels were normalized to GAPDH. (B) DANCR relative expression in Hs578T cells, MCF-7 cells, T47D cells, MDA-MB-468 cells and MDA-MB-231 cells. The expression of DANCR in Hs578T cells was used as the control. (C) DANCR relative expression in MDA-MB-231 cells transfected with Scramble siRNA (Scramble) or siRNA targeting DANCR (siDANCR). The expression of DANCR in MDA-MB-231 cells (Normal) was used as the control. (D) MTT assay was used to detect the relative growth rate in MDA-MB-231 cells transfected with Scramble or siDANCR. The proliferation rate in Scramble group was used as the control. (E) Transwell invasion assay was used to detect the invasive capability in MDA-MB-231 cells transfected with Scramble or siDANCR. Representative images (×200) are given in the graph. (F) Quantitative analysis of the invasion cells per well of a 24-well plate. Data are means±s.d.; *P<0.05; ^#^*P*<0.01.

To determine whether DANCR plays a pivotal role in TNBC, we explored the expression levels of lncRNA DANCR in 63 pairs of TNBC tissues and the adjacent normal tissues. As shown in the quantitative real-time PCR (qRT-PCR) data, our results revealed that DANCR was significantly upregulated in 47 TNBC tissues compared with their corresponding normal tissues (*P*<0.05) (Fig. 1A). We also classified all the TNBC patients into three groups according to the lncRNA DANCR expression level compared with the adjacent normal tissue, and found that of all the patients, 75% were of high DANCR expression and 11% were with no significant change, only 14% patients were of low DANCR level. These data suggested a possible correlation between the lncRNA DANCR expression and TNBC development and progression.

Four well-characterized breast cancer cell lines, MCF-7, T47D, MDA-MB-468 and MDA-MB-231 were investigated in this study, and the normal breast cell line Hs578Bst was used as the control. We first found that DANCR were overexpressed in all of the four breast cancer cell lines compared with in Hs578Bst cells, of them MCF-7 and T47D had about 10-folds of DANCR expression level, however MDA-MB-231 and MDA-MB-468 had over 50-folds of DANCR expression (Fig. 1B). The similar trends were confirmed when the DANCR mRNA levels were detected using a different endogenous housekeeping gene, ACTB (β-Actin) (data not shown). To investigate the biological effect of DANCR in TNBC cells, we chose MDA-MB-231 as a model for further study. The DANCR siRNA was transfected into MDA-MB-231 cells, and qRT-PCR showed this siRNA had a strong effect in decreasing the level of endogenous DANCR (Fig. 1C), this knockdown efficacy was also confirmed using a different endogenous control ACTB (β-Actin) (data not shown). As a result, DANCR knockdown decreased the MDA-MB-231 cell proliferation (Fig. 1D) and invasion (Fig. 1E,F), respectively. These data show that TNBC is associated with increased levels of DANCR in our patient population.

### Knockdown of DANCR decreased TNBC tumor growth in nude mice

**Fig. 2.**
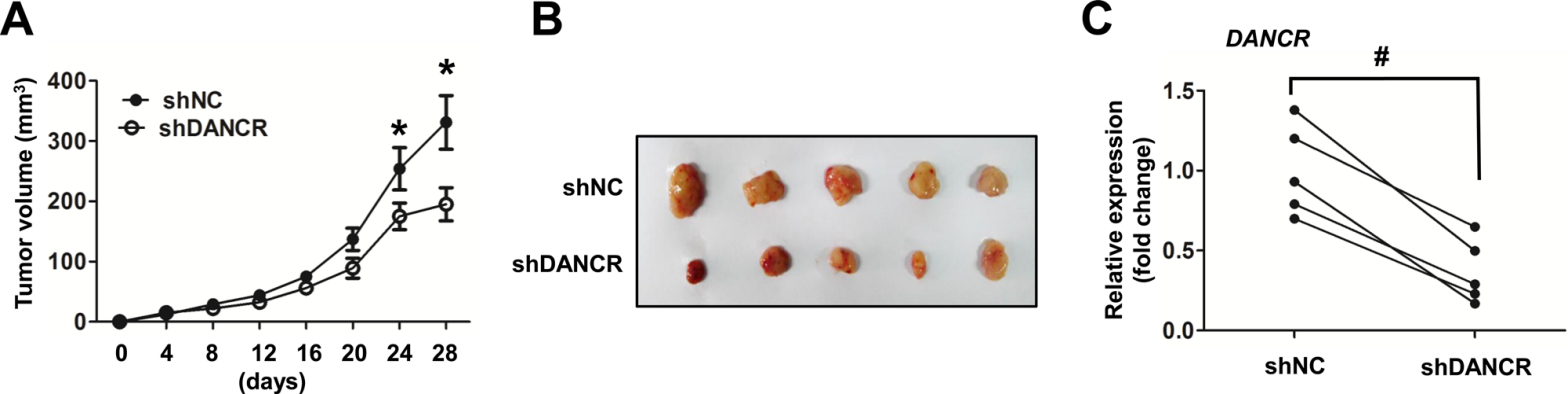
**Knockdown of DANCR decreased TNBC cells carcinogenesis in nude mice xenograft model.** (A) MDA-MB-231 were transduced with the indicated lentivirus encoding shRNA targeting DANCR (shDANCR) or a negative control shRNA (shNC). After puromycin selection, cells were injected subcutaneously into BALB/c female mice (*n*=5). Tumor volume was measured every 4 days. (B) 4 weeks after injection, mice were sacrificed and the tumors injected with the MDA-MB-231 cells were visualized. (C) LncRNA DANCR expression levels assessed by qRT-PCR in obtained tumor tissues between shNC group and shDANCR group. LncRNA DANCR expression levels were normalized to GAPDH. Data are means±s.d.; *P<0.05; ^#^*P*<0.01.

To investigate the effect of DANCR on breast cancer growth *in vivo*, MDA-MB-231 cells infected with lentivirus expressing shDANCR or shRNA non-target control (shNC) were injected orthotopically in the mammary gland of mice. The size of xenografts were monitored every 4 days and mice were sacrificed after 28 days. Our results showed that tumors generated from MDA-MB-231 cells infected with shDANCR grew significantly shower than the non-target control, either by the growth curve or by comparing the excited tissues (Fig. 2A,B). These results suggested that DANCR was capable of regulating breast tumor growth generated from MDA-MB-231 cells. Furthermore, we found that DANCR was successfully knocked down *in vivo* when the DANCR level in tissues from the xenografts generated from the MDA-MB-231 cells was infected with shNC or shDANCR (Fig. 2C). Altogether, these results confirm that under *in vivo* conditions, knockdown of DANCR inhibited the tumorigenic and development of TNBC cells.

### Knockdown of DANCR repressed TNBC cancer stem cell markers expression

**Fig. 3.**
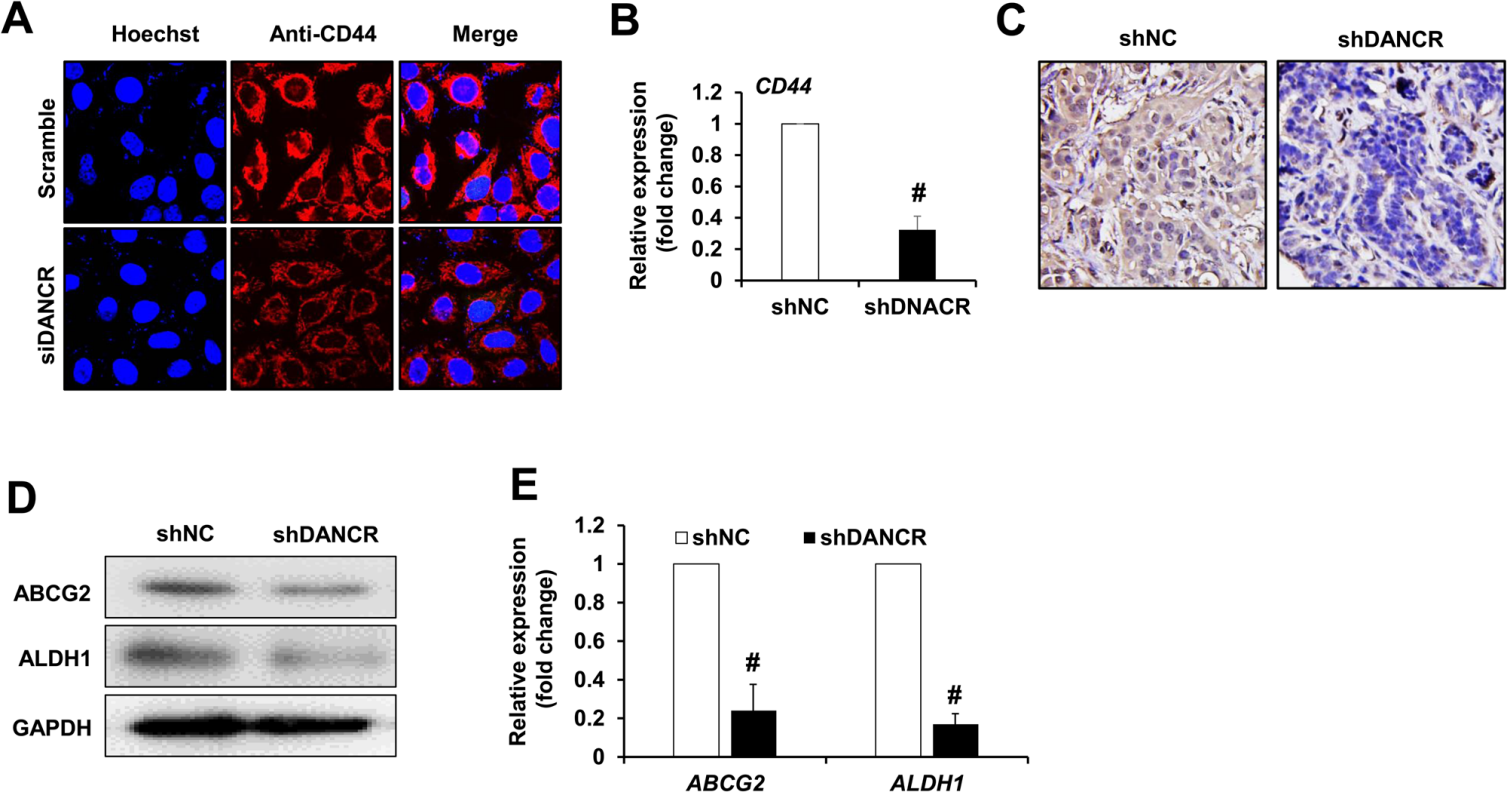
**DANCR repressed TNBC cancer stem cell marker expression.** (A) Immunofluorescence assay was used to detect the protein level of CD44 and CD24 in MDA-MB-231 cells transfected with Scramble or siDANCR. (B) Shown is real time PCR assay data for the expression of *CD44* gene in xenograft tissues generated from MDA-MD-231 cells infected with non-target control (shNC) or shDANCR (shDANCR). (C) Immunohistochemistry assay was used to detect the CD44 protein level in MDA-MB-231 cells infected with shNC or shDANCR. Representative images (200×) were given in the graph. (D) Western blot assay and (E) real time PCR assay were used to detect the protein level and mRNA of ABCG2 and ALDH1 in MDA-MB-231 cells transfected with Scramble or siDANCR, respectively. Data are means±s.d.; ^#^*P*<0.01.

Since DANCR knockdown inhibited cell proliferation and invasion of MDA-MB-231 cells, we further investigated the mechanism of how it regulates target genes. We examined the expression of CD44 and CD24 by immunofluorescence assay, and ABCG2, ALDH1 expression by western blot assay in DANCR knockdown MDA-MB-231 cells. We found that there was nearly no CD24 expression in either group; however, we found that DANCR knockdown significantly downregulated the protein level of CD44 in MDA-MB-231 cells *in vitro* when the CD44 protein level was detected using immunofluorescence staining (Fig. 3A), as well as the mRNA level using real time PCR assay (Fig. 3B). At the same time, negative control (shNC) or DANCR knockdown (shDANCR) MDA-MB-231 cells were injected orthotopically in the mammary gland in 8-week-old BALB/c female mice to establish the xenograft model, and xenograft tissues in nude mice were finally applied for immunochemistry staining, western blot and real time PCR assay. Our results indicated that the CD44 protein level was significantly reduced in the xenograft tissues (Fig. 3C). Moreover, we found the both the protein levels and mRNA levels of ABCG2 and ALDH1 were downregulated in the xenograft tumors generated from MDA-MB-231 cells transfected with shDANCR compared with shNC (Fig. 3D,E), whereas the expression of CD24 showed limited change (no significance, data not shown).

**Fig. 4.**
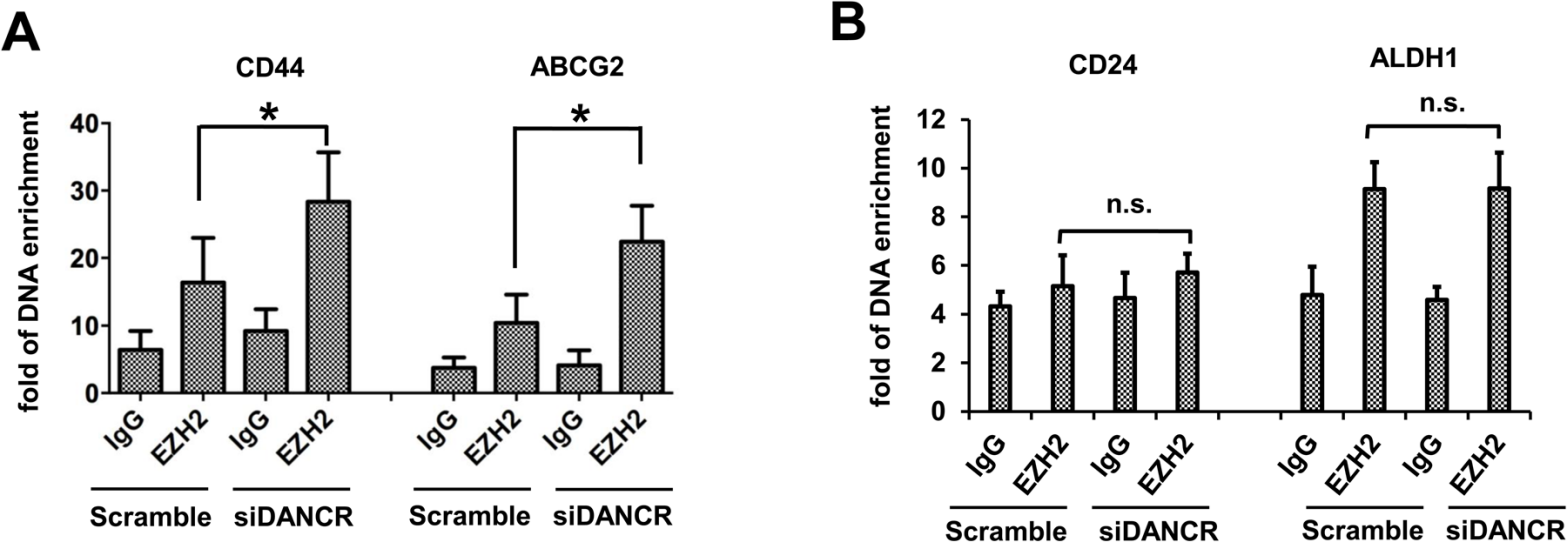
**LncRNA DANCR modulate the transcription of target genes through binding to promoters.** (A) ChIP assay was used to detect the enrichment of immunopreciated DNA level on the promoter of CD44 and ABCG2 genes in MDA-MB-231 cells transfected with Scramble or siDANCR. (B) ChIP assay was used to detect the enrichment of immunopreciated DNA level on the promoter of CD24 and ALDH1 genes in MDA-MB-231 cells transfected with Scramble or siDANCR. Data are means±s.d.; *P<0.05; n.s., not significant.

Considering the critical role of DANCR in prostate cancer invasion through modulating the binding of EZH2 on those gene promoters (Jia et al., 2016), we focused on how DANCR promotes the expression of these four genes. To verify whether the polycomb repressive complex 2 (PRC2) family member is an important way for lncRNAs to modulate the transcription of target genes, ChIP (chromatin immunoprecipitation) assay was applied. As shown in Fig. 4A, DANCR knockdown increased the binding of EZH2 on the *CD44* and *ABCG2* gene promoters in MDA-MB-231 cells. However, we did not find significant changes in the binding of EZH2 onto the *ALDH1* and *CD24* gene promoters due to DANCR knockdown since the enrichment of DNA had no significant differences compared with the IgG controls, respectively (*P*>0.05) (Fig. 4B).

### DANCR level was associated with tumor progression in TNBC patients

**Fig. 5.**
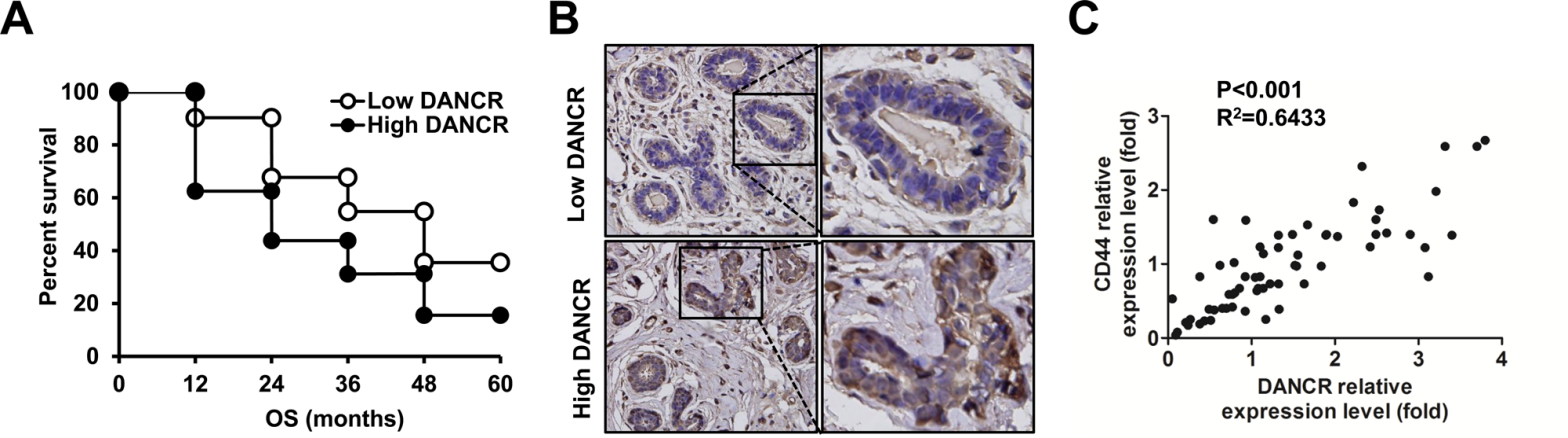
**Expression of DANCR was associated with tumor progression in TNBC patients.** (A) The correlation between lncRNA DANCR expression and the overall survival of TNBC patients. Kaplan–Meier analysis of overall survival was analyzed according to lncRNA DANCR expression levels. (B) Immunohistochemistry assay was used to detect the CD44 protein level in TNBC tissues between high DANCR expression and low DANCR expression. Representative images (200×) were given in the graph, magnification of the selected zones 400×. (C) Positive correlation between the expression of DANCR and CD44 in 63 TNBC samples. The relative expression of DANCR and CD44 was quantified using qPCR and normalized by GAPDH.

To further investigate the association of lncRNA DANCR with clinicopathological features of TNBC patients, the median value of lncRNA DANCR (2.94) in all TNBC tissues was used as a cutoff value, therefore all patients were divided into two groups: high DANCR expression group (≥2.75; *n*=32) and low DANCR expression group (<2.75; *n*=31). The relationship of lncRNA DANCR with various clinical features of TNBC was analyzed and was summarized in Table 1. The results showed that expression of lncRNA DANCR was significantly associated with TNM stages, histologic grade and lymph node metastasis (*P*<0.05). However, there was no significant correlation of DANCR expression with other clinical features such as age or tumor size (*P*>0.05). To further investigate the correlation of lncRNA DANCR expression with overall survival (OS) of TNBC patients, Kaplan–Meier analyses were performed. We found that overall survival time of the high lncRNA DANCR expression group was significantly shorter than that of the low lncRNA DANCR expression group (*P*<0.05, Fig. 5A). These results indicated that lncRNA DANCR expression may play an oncogenic role in breast cancer progression. Furthermore, we found that knockdown of DANCR decreased the expression of CD44 in the tissues from patient samples compared with their corresponding normal tissues, as detected by immunohistochemistry analysis (Fig. 5B). We further analyzed the expression of lncRNA DANCR and CD44 in clinical specimens with Pearson correlation analysis which showed a positive correlation between them (Fig. 5C).

**Table 1.**
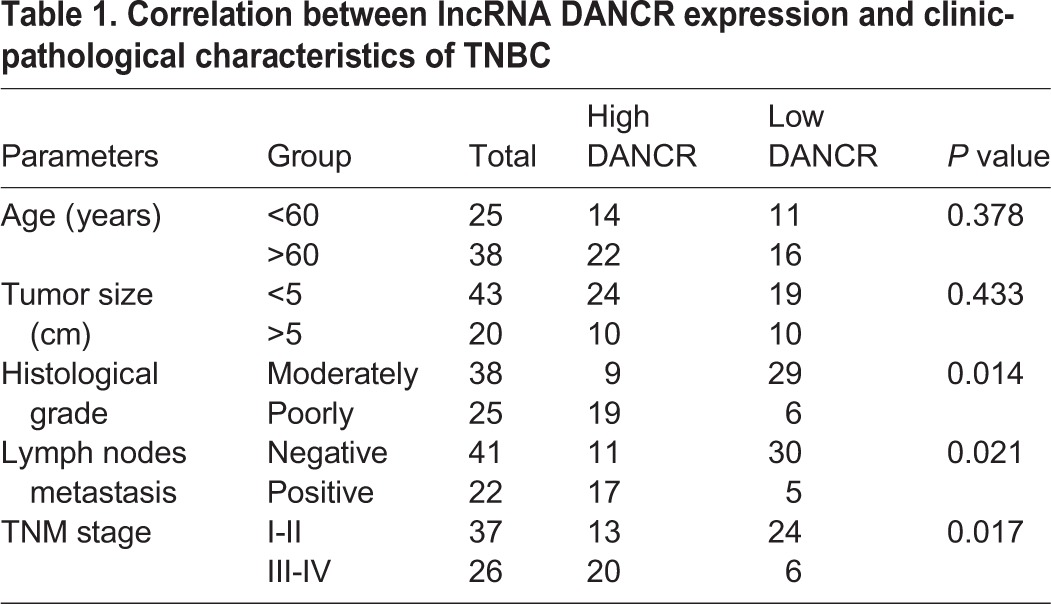
**Correlation between lncRNA DANCR expression and clinic-pathological characteristics of TNBC**

## DISCUSSION

Cancer cells from TNBC often display a profile of cell surface markers that are similar to that of breast cancer stem cell (BCSC), characterized by the phenotype CD44+/CD24− in which CD44 is expressed at high levels but levels of CD24 are low or undetectable (Yang et al., 2016; Honeth et al., 2008). In addition, ABCG2 alone can be considered a suitable marker for breast cancer, in particular for TNBC phenotype (Britton et al., 2012). Furthermore, ALDH1 expression was described to be higher in TNBC than non-TNBC cells (Li et al., 2013), and, in a small case of a series of TNBC patients, its expression was associated with poor clinical outcomes (Kim et al., 2014). Taken together, many observations suggest that targeting TNBC markers, such as CD44, CD24, ABCG2 or ALDH1, may be an effective strategy to treat TNBC (Opyrchal et al., 2014; Palasuberniam et al., 2015; Liu et al., 2015a).

Increasing evidence highlights that lncRNAs can serve as diagnostic biomarkers and therapeutic targets in solid tumors, including breast cancer (Zhang et al., 2013; Xiao et al., 2016); however, their relative expression levels in various subtypes of human breast cancer, particularly the TNBC subtype, remain unknown. Yuan et al. firstly identified DANCR, a lncRNA usually high expressed in cancers (Jia et al., 2016; Liu et al., 2015b), as a novel oncogene in hepatocellular carcinoma, and its activation might be a new characteristic of tumor cells with stemness features. Jia et al. reported that lncRNA DANCR expression increased in prostate cancer, moreover, DANCR promoted invasion and migration of prostate cancer cells *in vitro* and enhanced metastasis of xenograft prostate tumor in mouse model (Jia et al., 2016). In the present study, we used qRT-PCR to quantify the expression levels of DANCR in 63 pairs of TNBC subtype tissues, and we found that lncRNA DANCR expression increased in TNBC cancer tissues and TNBC cell lines. Moreover, knockdown of endogenous DANCR inhibited proliferation and invasion of MDA-MB-231 *in vitro* and enhanced tumorigenesis of xenograft breast cancer in mouse model.

Mechanically, Yuan et al. demonstrated that DANCR could regulate stabilization of mRNA, suggesting that the effect of DANCR on β-catenin reservoir might be independent from Wnt-signaling activation and carcinogenic mutation in exon3 of CTNNB1 (Yuan et al., 2016). Jia et al. found that TIMP2/3 were target genes of DANCR and knockdown of DANCR leads to up-regulation of TIMP2/3, and decreased binding of EZH2 and H3K27me3 on the promoter of TIMP2/3. Furthermore, they confirmed that DANCR repressed expression of TIMP2/3 synergistically with EZH2 (Jia et al., 2016).

In the present study, we found that knockdown of DANCR was associated with increased binding of EZH2 on the promoters of CD44 and ABCG2 and concomitant reduction of expression of these genes suggesting that they may be DANCR targets in TNBC. ALDH1 was also decreased after DANCR knockdown, although this could not be attributed to a change in EZH2 binding at its promoter.

Yuan et al. determined that DANCR upregulation was significantly associated with frequent tumor recurrence and cancer-related death and indicated that DANCR might be an attractive biomarker for risk prognostication, and that hepatocellular carcinoma (HCC) patients with DANCR overexpression should receive appropriate adjuvant therapies after hepatectomy (Yuan et al., 2016). We analyzed dysregulated DANCR expression and the clinicopathological characteristics of TNBC, and showed that expression of DANCR was significantly associated with TNM stage, histologic grade and lymph node metastasis.

In summary, our results indicate that knockdown of lncRNA DANCR inhibits proliferation, invasion and tumorigenesis of TNBC. DANCR could be a potential target for the treatment of breast cancer.

## MATERIALS AND METHODS

### Tissue specimens

The work described has been carried out in accordance with The Code of Ethics of the World Medical Association. A total of 63 fresh TNBC tissues and paired adjacent non-tumor tissues were obtained from patients who had undergone surgical resection of TNBC between 2009 and 2013 at the Department of General Surgery of Shanghai Jiaotong University Xinhua Hospital and the Shandong Haifushan Hospital. We clarified the TNBC patients into three groups according to the lncRNA DANCR relative expression. The normalized values ≤0.5 and ≥2.0 were used to determine low-expression and high-expression of DANCR expression, respectively, as reported previously (Goto et al., 2006; Cizkova et al., 2013). The relative expression of DANCR with T/N>2 was recognized as high-expression, T/N<0.5 was recognized as low-expression, 0.5<T/N<2 was recognized as unchanged, where the T/N means the ratio of relative expression of tumor tissues/non-tumor tissues. No patients had been treated with radiotherapy or chemotherapy before surgery. This study was approved by the Ethics Committee of Peking University Health Science Center, and informed consent was obtained from each patient involved in the study.

### Immunohistochemistry

Paraffin-embedded tissue was sectioned in 3 μm slices. After removal of the paraffin and antigen retrieval in citrate buffer (pH 6.0) at 100°C for 5 min, slices were incubated overnight with anti-CD44 antibody (1:100, Abcam, Shanghai, China) at 4°C. Detection was performed in an automated slide staining instrument (Ventana Medical Systems, Tucson, AZ, USA) by using the iView DAB staining kit (Ventana Medical Systems, Tucson, AZ, USA) and the slices were counterstained by hematoxylin.

### Cell culture and transfection

Human normal breast cancer cell line Hs578Bst cell and four well-characterized breast cancer cell lines, MCF-7, T47D, MDA-MB-468 and MDA-MB-231 cell lines (ATCC^®^ HTB-26™) were purchased from the American Type Culture Collection (ATCC, Shanghai, China). MDA-MB-231 cells were cultured in DMEM supplemented with 1 mM sodium pyruvate, 100 U/ml penicillin, 100 μg/ml streptomycin and 10% fetal bovine serum. Cells achieved 70-80% confluence for lentiviral infection or 30-50% confluence for siRNA transfection using Lipofectamine RNAiMax reagent (Invitrogen, Carlsbad, CA, USA). DANCR knockdown in MDA-MB-231 cells was carried out by transfecting siRNAs targeting DANCR (Suzhou Ribo Life Science, Suzhou, China). Briefly, cells were transfected with Scramble control or siRNAs (50 nM) using Lipofectamine RNAiMax reagent (Invitrogen, Carlsbad, CA, USA). The sequence of siRNA for DANCR and Scramble were: siDANCR, sense 5′-GGCCAAAUAUGCGUACUAAUU-3′, antisense 3′-UUCCGGUUUAUACGCAUGAUU-5′; Scramble sense, 5′-CGUACUAAGGCCAAAUAUGUU-3′, antisense, 3′-UUGCAUGAUUCCGGUUUAUAC-5′. DANCR knockdown in MDA-MB-231 cells for xenografts was carried out by LV5 lentiviral vectors encoding short hairpin RNA (shRNA) targeting non-specific control (NC) or human Lnc RNA DANCR (5′-GGAGCTAGAGCAGTGACAATG-3′), which were constructed by GenPharma (Shanghai, China).

### RNA isolation and qRT-PCR

Total RNA was isolated from TNBC tissues or cells using Trizol reagent (Invitrogen, Carlsbad, CA, USA) according to the manufacturer's instructions. The expression level of DANCR in TNBC tissues and cell lines was measured by qRT-PCR using the SYBR-Green method (Takara, Dalian, China) according to the manufacturer's protocol and normalized with GAPDH or ACTB (β-Actin). The primers were as follows: *DANCR* sense: 5′-GCCACAGGAGCTAGAGCAGT-3′; *DANCR* antisense: 5′-GCAGAGTATTCAGGGTAAGGGT-3′; *GAPDH* sense: 5′-AACGGATTTGGTCGTATTGGG-3′; *GAPDH* antisense: 5′-CGCTCCTGGAAGATGGTGAT-3′. *ACTB* sense: 5′-TCCTGGGCATGGAGTCCTGT-3′; anti-sense: 5′-TCGGCAATGCCAGGGTACAT-3′. All experiments were performed using the 2^−ΔΔCt^ method (Arocho et al., 2006). Each experiment was performed in triplicate.

### Western blot analysis

Cells were lysated with 1×RIPA buffer (Cell Signaling Technology, Danvers, MA, USA) and analyzed by western blot assay. Primary antibodies including ABCG2 antibody (Cat No. 58222), ALDH1 antibody (Cat No. 166362) and GAPDH (Cat No. 25778) were all from Santa Cruz Biotechnology (Dallas, Texas, USA). Secondary anti-mouse or anti-rabbit IgG were supplied by Zhongshanjinqiao Biotech (Beijing, China), and the final signal was detected using the UVP Imaging.

### Proliferation assay

For proliferation assays, 1×10^4^ MDA-MB-231 cells (Scramble and DANCR knockdown) were plated in 96-well plates. After 3 days MTT solution was added to wells (0.5 mg/ml). Cells were incubated for 4 h at 37°C. Media was removed and MTT formazan crystals were solubilized in DMSO. Absorbance was measured at 560 nm in a microplate reader (Bio Rad, Martinez, CA, USA).

### Cell invasion assay

Transwell invasion assay was performed in 8 μm-pore transwell inserts (Millipore, Bedford, MA, USA). For *in vitro* invasion assays, the upper chambers of transwell were pre-coated with diluted matrigel (BD Biosciences, Sparks, MD). 1×10^5^ cells were seeded onto upper chamber in serum-free medium and medium containing 10% serum were added to the lower chamber as a chemoattractant. After incubation for 24 h, the upper surface of the insert was wiped with a cotton swab and cells that migrated to the lower surface were fixed by 4% paraformaldehyde and stained with crystal violet. Cell numbers were counted in 6 random fields per well.

### Immunofluorescence assay

Briefly, MDA-MB-231 cells were seeded on coverslips in 35 mm dishes and treated with siRNA respectively for 24 h, then cells were fixed with 4% paraformaldehyde. Fixed cells were incubated with anti-CD44 (1:100, Cat No. 6124) and CD24 antibodies (1:100, Cat No. 64064) (Abcam, Hangzhou, China) and then the fluorochrome-tagged secondary antibody (1:500, FITC-conjugated anti-rabbit, TRITC-conjugated anti-mouse) (Abcam, Hangzhou, China). Following stained with Hoechst33342 in PBS buffer, coverslips were mounted on slides.

### Xenografts

This study was approved by the Ethics Committee of Peking University Health Science Center and complied with the ARRIVE guidelines. MDA-MB-231 cells (1×10^6^ cells/mouse) were injected orthotopically in the mammary gland in 8-week-old BALB/c females (*n*=5 per experimental group). The mice were fed *ad libitum*. Tumor latency and growth was measured. Tumor volumes were calculated as ellipsoids (D×d^2^/2) by measuring the main diameter (D) and the smaller diameter (d) and plotted versus time (days). For experiments with post-surgery adjuvant treatments, primary tumors were surgically removed when the tumor volume was ∼300 mm^3^.

### Statistical analysis

All statistical analyses were performed using SPSS version 13.0 software. The measurement data were analyzed by one-way ANOVA. Randomized block design ANOVA was used to analyze the statistical difference among different tissue types. Survival curves were plotted using the Kaplan–Meier method, and differences between survival curves were tested using the log-rank test. All data are presented as the mean±s.d. from at least three independent experiments. *P*<0.05 was considered statistically significant.
